# Hepatitis E Virus Shows More Genomic Alterations in Cell Culture than In Vivo

**DOI:** 10.3390/pathogens8040255

**Published:** 2019-11-22

**Authors:** Gulce Sari, Martijn D.B. van de Garde, Anne van Schoonhoven, Jolanda J.C. Voermans, Annemiek A. van der Eijk, Robert A. de Man, Andre Boonstra, Thomas Vanwolleghem, Suzan D. Pas

**Affiliations:** 1 Department of Gastroenterology and Hepatology, Erasmus University Medical Center, Postbus 2040 3000 CA Rotterdam, The Netherlands; g.sari@erasmusmc.nl (G.S.); Anne.Vs@live.nl (A.v.S.); r.deman@erasmusmc.nl (R.A.d.M.); p.a.boonstra@erasmusmc.nl (A.B.); 2 Department of Viroscience, Erasmus University Medical Center, Postbus 2040 3000 CA Rotterdam, The Netherlandsa.vandereijk@erasmusmc.nl (A.A.v.d.E.); s.pas@bravis.nl (S.D.P.); 3 Laboratory of Experimental Medicine and Pediatrics, Faculty of Medicine and Health Sciences, University of Antwerp and Department of Gastroenterology and Hepatology, Antwerp University Hospital, 10 2650 Antwerp, Belgium; 4 Microvida, Location Bravis Roosendaal, Molengracht 21 4818 CK Breda, The Netherlands

**Keywords:** Hepatitis E virus, HEV whole genome sequencing, viral adaptation, spontaneous mutagenesis

## Abstract

Hepatitis E Virus (HEV) mutations following ribavirin treatment have been associated with treatment non-response and viral persistence, but spontaneous occurring genomic variations have been less well characterized. We here set out to study the HEV genome composition in 2 patient sample types and 2 infection models. Near full HEV genome Sanger sequences of serum- and feces-derived HEV from two chronic HEV genotype 3 (gt3) patients were obtained. In addition, viruses were sequenced after in vitro or in vivo expansion on A549 cells or a humanized mouse model, respectively. We show that HEV acquired 19 nucleotide mutations, of which 7 nonsynonymous amino acids changes located in Open Reading Frame 1 (ORF1), ORF2, and ORF3 coding regions, after prolonged in vitro culture. In vivo passage resulted in selection of 8 nucleotide mutations with 2 altered amino acids in the X domain and Poly-proline region of ORF1. Intra-patient comparison of feces- and serum-derived HEV gt3 of two patients showed 7 and 2 nucleotide mutations with 2 and 0 amino acid changes, respectively. Overall, the number of genomic alterations was up to 1.25× per 1000 nucleotides or amino acids in in vivo samples, and up to 2.84× after in vitro expansion of the same clinical HEV strain. In vitro replication of a clinical HEV strain is therefore associated with more mutations, compared to the minor HEV genomic alterations seen after passage of the same strain in an immune deficient humanized mouse; as well as in feces and blood of 2 immunosuppressed chronically infected HEV patients. These data suggest that HEV infected humanized mice more closely reflect the HEV biology seen in solid organ transplant recipients.

## 1. Introduction

The hepatitis E virus (HEV) is a non-enveloped positive-sense single-stranded RNA virus, belonging to the family *Hepeviridae* within the genus *Orthohepevirus* [1]. The genome of HEV is approximately 7.2 kb long and contains three open reading frames (ORF). ORF1 encodes a non-structural protein which contains a methyltransferase, a Y-domain, a papain like cysteine protease, a helicase, and a RNA-dependent RNA polymerase. ORF2 encodes for structural proteins forming the viral capsid. ORF3 overlaps with ORF2 and encodes a viroporin essential for the release of infectious particles [2,3]. 

In an infected host, a virus can compartmentalize in different locations and tissues, resulting in intra-host viral variants. Genomic viral variants of hepatitis C virus, human immunodeficiency virus, and Epstein bar virus have been observed in different compartments with altered viral entry, viral replication, and treatment response [4,5,6,7,8,9]. In addition to the liver, HEV is also found in blood and feces of infected patients. The detection of HEV RNA in feces is of clinical importance, as it has been shown to predict viral relapse after ribavirin (RBV) treatment cessation upon undetectable serum/plasma viral titers in chronic HEV patients [10,11]. However, apart from emergent viral variants following RBV treatment [12], little is known on HEV’s spontaneous mutational drift or compartmentalization.

An expanding breadth of in vitro and in vivo models are available to study the biology of HEV, each with specific strengths and weaknesses. Permissive cell lines exist for clinical HEV isolates obtained from feces of infected patients, such as A549, PLC/PRF-5, HepG2, Huh7, and their sub clones [13,14]. However, these cell-lines are often deficient in intracellular interferon responses and may substantially differ from differentiated human hepatocytes. An alternative in vivo model consists of humanized mice, in which the mouse liver is repopulated with fully functional human hepatocytes. We and others showed that the HEV infection course seen in chronically infected patients can be mirrored in this system, with the advantage of studying viral shedding into blood and feces, in addition to HEV propagation, in an immune-compromised host [15,16,17]. 

With the current study our aim is to examine whether in vitro and in vivo passage in 2 frequently applied model systems for HEV would result in a dominant mutant hepatitis E viral strain. In addition, we examined compartmentalization of HEV by comparing genomes of serum- and feces-derived HEV from two chronic HEV gt3 patients at different time points of their infection course. As we do not focus on viral diversity per se in the examined compartments or models, we applied Sanger sequencing for our research questions. 

## 2. Results

### 2.1. Feces- and Serum-Derived HEV Sequences Show Mostly Synonymous Mutations

**Figure 1:**
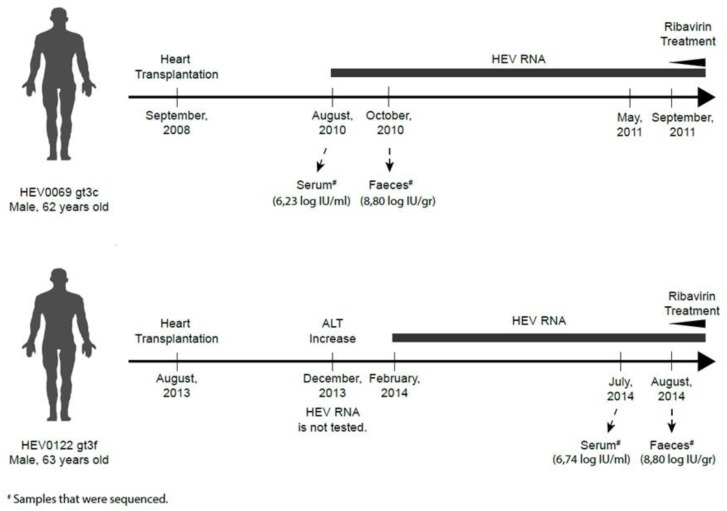
Schematic representation of two chronic Hepatitis E Virus (HEV) genotype 3 (gt3) cases. Clinical samples were obtained from two male heart transplant recipients: HEV0069 gt3c and HEV0122 gt3f. Sample collection was done before treatment at either the Erasmus Medical Centre or the University Hospital Antwerp. Both patients had detectable HEV RNA in their EDTA-plasma for more than 3 months (defined as chronic HEV infection).

**Figure 2:**
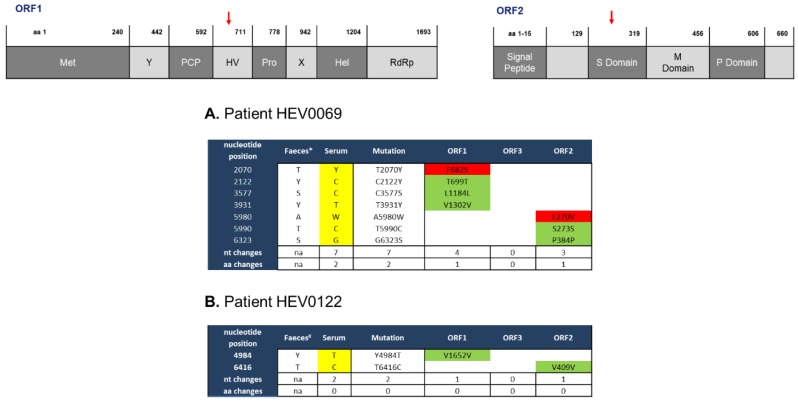
Limited number of intra-patient alterations in HEV genomes isolated from serum and feces. Intra-patient comparison of HEV genomes isolated from serum and feces of two chronic HEV patients (HEV0069 (**A**) and HEV0122 (**B**)) was performed. The feces isolate is used as reference sequence; the serum isolate is aligned to the feces isolate sequences and differences are highlighted. nt, nucleotide; aa, amino acid; na, not applicable; ORF, open reading frame; Green, no change in amino acid sequence; Red, change in amino acid sequence; Yellow, nucleotide differences with respect to feces nucleotide sequence. Red arrows point the domains with amino acid change. Nucleotide abbreviations; M = A,C; S = G,C; W = A,T; Y = T,C. The HEV wbGER27 (* GenBank accession number: FJ705359) and the HEV RKI (#Genbank accession no. FJ956757.1) are used for sequence alignments of HEV0069 and HEV0122 respectively.

The analysis of both serum/plasma- and feces-derived HEV is essential in the care for chronically infected HEV patients [10], but little is known about the spontaneous mutations of HEV in these compartments. We therefore compared the genomic alterations of feces- and serum-derived virions obtained from two heart-transplanted patients during different time points of their chronic HEV infection course (Figure 1). After PCR optimizations, sequenced HEV0069 feces and serum samples resulted in a HEV genomic coverage of 98.3–100%. For HEV0122 a genomic coverage of 85.4–88.9% was obtained (Supp. Table S2). Feces and serum samples of HEV0069 were obtained before the chronic infection was established (within the first 2 months after diagnosis, [10]). Intra-patient comparison of feces and serum HEV0069 isolates revealed 5 synonymous, 3 in ORF1 (Y2122C, S3577C, Y3931T) and 2 in ORF2 (T5990C, S6323S), and 2 non-synonymous mutations, 1 in ORF1 Hypervariable region (Y2070T) and 1 in ORF2 Shell Domain (A5980W) (Figure 2A). HEV sequences derived from feces and serum from patient HEV0122 revealed only 2 synonymous changes, 1 in ORF1 (Y4984T) and 1 in ORF2 (T6416C) (Figure 2B). The number of genomic alterations at the nucleotide level between both compartments was 0.974 nucleotide changes per 1000 nucleotides in HEV0069 at the acute phase of his HEV infection and 0.311 nucleotide changes per 1000 nucleotides in HEV0122, at the chronic phase of his HEV infection. In both patients the sampling interval between serum and feces was 1 to 2 months, which may have an influence on the observed number of mutations. However, a lower genomic alteration between both compartments is observed during the later stage of infection, i.e., after 5–6 months of HEV RNA positivity in patient HEV0122. This suggests that there is no progressive genetic drift in the feces compartment. Indeed, HEV0069 samples were obtained at HEV diagnosis, when initial high viral replication rates in the liver may have yielded a higher number of genomic alterations.

### 2.2. HEV Adapts to Cell Culture with 7 Nonsynonymous Nucleotide Changes during In Vivo Passage

**Figure 3:**
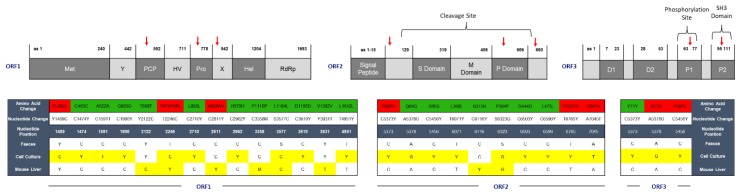
HEV adapts to in vitro culture but shows limited mutagenesis in the humanized mouse model. Genomic differences after in vitro and in vivo passage of HEV derived from HEV0069 feces are depicted per ORF. The HEV0069 feces isolate is used as reference sequence and the nucleotide and amino acid sequence differences are highlighted in yellow and red, respectively. nt, nucleotide; aa, amino acid; ORF, open reading frame; Green, no change in amino acid sequence; Red, change in amino acid sequence; Yellow, nucleotide differences with respect to feces nucleotide sequence. Nucleotide abbreviations; M = A,C; S = G,C; W = A,T; Y = T,C. HEV0069 sequence is relative to HEV wbGER27 (GenBank accession number: FJ705359).

To further assess the mutational drift of HEV gt3 in vitro and in vivo, A549 cells and a human-liver chimeric mouse were independently inoculated with HEV derived from feces of patient HEV0069. Infection was maintained for 7 passages onto A549 cells and culture supernatant was harvested 10 days after the last passage, covering a total of 111 days of in vitro propagation [15]. The culture supernatant was sequenced and showed 19 single nucleotide mutations compared to the initial HEV0069 feces-derived HEV inoculum (Figure 3). Seven of these mutations resulted in amino acid substitutions; FL462L, W741R in ORF1, P68S, E270V, Y532D, D625V in ORF2 and N73S, P99L in ORF3. The amino acid substitution in ORF2 resulted in an alteration in the P domain of ORF2 and 2 alterations in ORF3 coding regions, P1 and SH3 domains respectively. 

**Figure 4:**
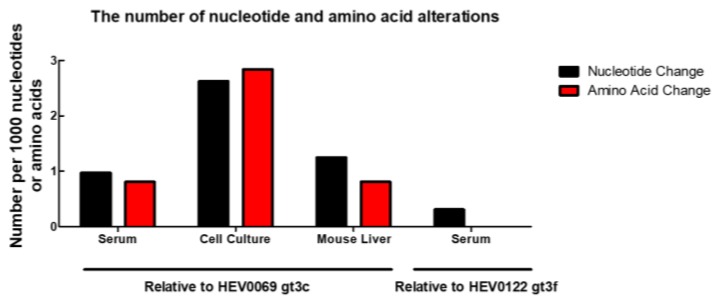
Frequency of alterations was highest after in vitro cell culture. The number of mutations in isolates obtained from serum, cell culture, and human liver chimeric mouse liver relative to the sequence derived from the feces isolate. The number of variations was divided by the total sequence length of the genome or the expected protein sequence length and then normalized to 1000 nucleotides or amino acids.

HEV inoculations in the human-liver chimeric mouse model result in a stable chronic infection, similar to what is seen in solid organ transplant recipients [15,18]. To assess the genetic drift in a correlate for an immunocompromised host, a mouse with humanized liver was infected with feces-derived HEV0069 for 6 weeks, which is comparable to the serum and feces sampling interval of patients. The sequenced HEV genome, isolated from mouse liver after 6 weeks of infection showed 8 single nucleotide mutations, and only 2 of these mutations resulted in amino acid alterations: W741WR and A929AV in ORF1, the poly-proline region and macro domain respectively (Figure 4). The HEV mutation frequency in the humanized mouse model over 6-week infection time is comparable to the number of HEV’s genomic alterations seen at different compartments (serum and feces) of the same patient, but lower to what we observe after in vitro propagation (Figure 4). Our data therefore suggests that this clinical HEV strain adapts to in vitro culture in A549 cells, but shows minimal changes in an experimental immune compromised host with differentiated human hepatocytes.

## 3. Discussion

Chronic HEV gt3 infections are a problem in immunocompromised hosts in Europe, with no approved treatment available. Both RBV and interferon are used as treatment option [10]. The first HEV clinical practice guidelines advocate to analyse both patient serum as feces for the presence of HEV RNA, as viral relapse may occur when HEV RNA remains detectable in feces upon RBV treatment withdrawal [10,11]. Point mutations in serum-derived HEV have been shown to enhance HEV replication associated with clinical RBV resistance [13,14,15,19,20,21,22,23,24,25,26,27], but little is known on the spontaneous mutations between both compartments. 

We here performed intra-patient comparison of full genome sequences of HEV gt3 isolates from serum and feces of two chronic HEV patients and further examined the genetic drift of 1 of these clinical HEV strains in 2 established in vitro and in vivo model systems. Our data suggest that compared to in vitro replication, in vivo selection pressure in an experimental immune deficient host or between feces and blood of 2 immunosuppressed heart transplant patients is relatively small.

Intra-patient HEV nucleotide sequences showed 5 synonymous and 2 nonsynonymous differences between feces and serum isolates from one patient early after his HEV diagnosis, whereas only 2 synonymous mutations were found in another patient after at least 5 months of ongoing HEV replication. We were unable to sequence a 600 bp region located between nt position 5346–5951 for HEV0122 serum and a 850 bp region between nt position 5097–5951 for HEV0122 feces. For HEV0069, for which near complete sequences were obtained, we did not observe any mutation between nt position 3931 and 5980 or above 6323, corresponding to the non-sequenced part of HEV0122. One of the nonsynonymous alterations observed for the first patient (HEV0069) is located in the S domain of ORF2. Although the feces and serum sampling time during their chronic HEV infection course differed, the genomic alteration ratio was lower at a later stage of infection (patient HEV0122). This would argue against an important genetic drift between serum and feces in a chronically HEV-infected individual. Most probably, a higher replication and selection is observed during the initial stage of the infection with ongoing spread in the liver, due to the relative high mutation rates of RNA viruses [28]. On the other hand, if the infection is established for a longer period, as seen in patient HEV0122, the fittest quasispecies population would have been selected and persisted, resulting in a lower number of variants. 

In addition to inter- and intra-patient comparison of 2 serum- and feces-derived HEV gt3 strains, the genomic alterations of 1 clinical HEV strain after in vitro and in vivo passage were assessed. Humanized mice showed lower viral titres in serum compared to feces and liver [15], which affect sequencing success due to low RNA yields. Therefore, we aimed to compare HEV RNA sequences of the inoculum and liver isolates obtained from HEV infected human-liver chimeric mouse. In this model immune selection pressure is absent, due to a maturation deficit in T, B, and NK cells, which allows the comparison of HEV mutagenesis during replication in the A549 cell-line in vitro and in differentiated hepatocytes in vivo. An 8 nucleotide-different viral variant and 2 amino acid alterations were identified in humanized mouse liver 6 weeks after inoculation. Two nonsynonymous alterations resulted in mutations in the poly-proline region and X domains of ORF1. The absence of immune cells may explain the relatively low mutational drift of HEV in this experimental immunocompromised host. In addition, we recently showed no induction of intra-cellular innate immune responses in HEV infected human hepatocytes, minimizing the intra-cellular immune pressure [18]. 

In the in vitro model, seven out of nineteen mutations identified after HEV culture on A549 cells resulted in amino acid changes. The amino acid changes affecting ORF1, FL462L, and W741R, are located in the N terminal region, which was reported to interact with intracellular signalling molecules in vitro [29], and therefore may affect host-virus interactions. In addition, in vitro serial passaging, but not in vivo replication in a humanized mouse model, resulted in alterations in HEV capsid protein’s amino acid sequence (P domain) of ORF2, which might impact antibody-mediated neutralization and P1 and SH3 domains of ORF3, possibly impacting intracellular signalling and trafficking. Further analysis is, however, necessary to determine if the found amino acid alterations also alter the protein structure. 

Upon comparison of in vitro and in vivo infection models, we demonstrate a higher mutation frequency in the supernatant of A549 cells compared to the chimeric mouse liver inoculated with the identical clinical strain. We have previously described a very fast viral expansion and plateau phase within 1–2 weeks after inoculation in vivo, while this may take up to 30 days and longer in vitro [15,18]. We therefore chose to sample chimeric liver after 6 weeks of in vivo replication and supernatant after 111 days of in vitro propagation, which would allow for the initial slower in vitro expansion compared to the in vivo model. Besides differences in viral kinetics, the observed genomic alterations after in vitro propagation and in vivo infection in humanized mice illustrate important model differences. Emergence of several nucleotide substitutions can affect the biology of HEV and alter its responsiveness to treatment. The knowledge of HEV’s genomic stability in different infection models therefore has important implications for its translation to the clinical situation. Based on our results, the in vivo model seems more representative for a genuine clinical HEV infection, with relatively low mutagenesis after 6 weeks of infection similar to the genomic alterations between serum and feces viral isolates. Whether the in vitro acquired nucleotide substitutions are due to the in vitro setting or the adaptation of the virus to a non-hepatic cell line remains to be determined.

All obtained HEV gt3 sequences contained double peaks that were verified by multiple forward and reverse reads, suggesting the presence of multiple viral populations within a single isolate. This phenomenon of intra-host heterogeneity has been previously described for HEV [20,27]. Viral variants that comprise at least 20% of the total viral population can be detected with the Sanger sequencing method we applied here, which can be a limitation of our study. Whether minority populations exist and may impact infectivity differences remains to be determined, using NGS. As we were restricted to samples with a high viral load for Sanger sequencing, we could not expand our observations to more biological replicates. Indeed, both sequenced experimental samples had HEV RNA levels of at least 7 log IU/mL or 7 log IU/gr tissue. In addition, these samples were amongst the highest available for the respective model systems (cfr Figure S1). The number of samples studied does not allow a statistical analysis and calls for more full-length HEV sequencing studies of both experimental and clinical samples to generalise these observations. 

In conclusion, our data suggest that viral compartmentalization is minimal between serum and feces in 2 chronic HEV patients. In vitro replication of a clinical HEV strain is associated with more mutations, compared to the minor HEV genomic alterations seen after passage of the same strain in an immune deficient humanized mouse. These data suggest that HEV infected humanized mice more closely reflect the HEV biology seen in solid organ transplant recipients. 

## 4. Materials and Methods 

### 4.1. Virus Isolates

Clinical samples were obtained from two heart transplant recipients (HEV0069 and HEV0122) [15], collected during the early and late chronic phase of their infection respectively, but before start of ribavirin treatment at either the Erasmus Medical Centre, the Netherlands or the University Hospital Antwerp, Belgium (Ethical Committee approval: 2012-522 and 15/21/227). Both patients had detectable HEV RNA (genotype 3c and 3f, respectively, [19]) in their EDTA-plasma for more than 3 months (Figure 1 and Figure S1) [30]. Transplantation immunosuppression regime of patient HEV0069 can be found in ref. 19 and Figure 4 and patient HEV0122 received tacrolimus, mycophenalate and methylprednisolone.

### 4.2. Cell Culture

The human lung adenocarcinoma cell line *A549* was cultured according to manufacturer’s recommendations. Briefly, cells were cultured in growth medium containing Dulbecco’s modified Eagle’s medium (DMEM; Lonza) and supplemented with 10% fetal bovine serum (FBS; Greiner Bio-one, Kremsmünster, Austria), 0.08% NaHCO3, 2 mM l-glutamine (Lonza), 1% penicillin-streptomycin (pen-strep; Lonza), and 0.5 μg/μL amphotericin B (Pharmacy, Erasmus Medical Center, Rotterdam, Netherlands). Cells were infected with the feces-derived HEV inoculum as described before [15]. In vitro derived HEV viruses were obtained from cell culture supernatant after seven passages of HEV0069 feces onto *A549* cells. 

### 4.3. Mouse Origin and In Vivo Experiments

Urokinase-type plasminogen activator (uPA) NOD/Shi-scid/IL-2Rɣ^null^(NOG) mice were bred at the Central Animal Facility of the Erasmus Medical Center (DEC nr 141-12-11) and offspring zygosity was determined using a copy number duplex qPCR performed on phenol-chloroform-isoamyl alcohol (Sigma-Aldrich, St. Louis, MO, USA)-extracted genomic mouse DNA from toe snip. TaqMan Genotyping master mix (Life technologies, Carlsbad, CA, USA), TaqMan uPA genotyping assay (Mm00422051_cn; Life Technologies) and Tert gene references mix (Life technologies) were used according to the manufacturer’s protocol. Homozygous uPA+/+ mice were transplanted with 0.5 × 10^6^ to 2 × 10^6^ viable commercially available cryopreserved human hepatocytes (Lonza, Basel, Switzerland, and Corning, Corning, NY, USA) via the intrasplenic injection route [31]. Hepatocyte engraftment level was determined using a human albumin ELISA as previously described (Bethyl laboratories, Montgomery, TX, USA) [31]. A successfully engrafted mouse was intravenously infected with feces-derived HEV0069 inoculum. HEV infection was repetitively demonstrated in weekly collected mouse fecal samples until sacrifice at week 6 using an ISO15189:2012-validated, internally controlled q RT-PCR, as described previously [32]. Chimeric mouse liver derived HEV RNA was isolated from a liver fragment obtained at sacrifice 6 weeks after HEV inoculation [15]. All studies including animals were approved by the animal ethics committee of the Erasmus University Medical Centre and conducted according to Dutch national guidelines. 

### 4.4. Viral RNA isolation and cDNA Synthesis

HEV RNA was isolated using the QIAamp Viral RNA Mini kit (Qiagen) according to manufacturer’s instructions andeluted in 2 × 30 µL elution buffer.

cDNA of HEV0069 feces, serum, human liver chimeric mouse liver and in vitro culture supernatant was synthesized using 1 µM HEV-specific primer Rv4, Rv8, Rv12 and Rv15 (Supp. Table S1A), 0.5 mM dNTP nucleotide mix (Roche) and 22 µL HEV RNA. Before cDNA synthesis potential secondary RNA structures were removed by heating to 65 °C for 5 min followed by 1 minute of incubation on ice. Subsequently, 80 U RNasin (Promega), 0.1 mM Dithiothreitol (DTT) (Invitrogen), 5× First Strand buffer (Invitrogen), and 400 U SuperscriptIII RT (Invitrogen) were added to a total volume of 40 µL and incubated for 60 min at 50 °C. HEV0122 feces and serum HEV RNA were made into cDNA with a Transcriptor First Strand cDNA Synthesis kit (Roche) following manufacturer’s instructions, with 10 µL HEV RNA input for each 20 µL reaction, using a combination of random hexamers and an oligo-dT primer.

### 4.5. PCR Amplification

HEV primer design was based on gt3 genomes with accession numbers FJ705359 and FJ956757.1 [32]. cDNA of HEV0069 infected samples were used to generate seven overlapping amplicons of ~2000 bp length each (Figure S2). Subsequently, two or three 500 bp long amplicons, overlapping ~50–100 bp, per primary product were generated using nested primers to cover the full HEV genome (Table S1A,B). Each 50µL reaction contained 10 µL HEV cDNA, 0.4 µM primers, 1× PCR buffer (Qiagen), 1 mM MgCl_2_ (Qiagen), 0.2 mM dNTP mix (Roche), and 2.5 U HotStar polymerase (Qiagen). For PCR, the conditions were 15 min 95 °C, 40 cycles of 1 min 95 °C, 1 min 50 °C, 3 min 72 °C, and 10 min 72 °C. 2 μL first PCR product served as template for the nested PCR. The PCR conditions were identical to first round PCR, with 40 cycles of 30 sec 95 °C, 30 sec 50 °C, and 1 min 72 °C. Amplicon 15 was amplified using an altered annealing temperature of 45 °C instead of 50 °C.

In order to amplify cDNA from serum and feces-derived HEV0122, additional primer pairs were adapted from Munoz-Chimeno et al. [33] as shown in Table S1C,D. PCR conditions were identical as described above, with minor modifications: 5 μL cDNA was used as input for the first round PCR, and 2.5 μL first PCR product served as template for nested PCR. The annealing temperatures during the PCR protocols were adjusted to match each primer set. 

### 4.6. Fragment Detection and Purification 

Presence and purity of the nested amplicons was assessed by gel electrophoresis on a 2% agarose gel and extracted using the GeneJET MinElute kit (Thermo Fisher) according to manufacturer’s instructions. UV exposure was kept to minimum. DNA concentrations were determined using Nanodrop (NanoDrop™2000, Thermo Fischer), and samples were diluted to 0.5 ng/µL–20 ng/µL. 

### 4.7. Sanger Sequencing

Sequencing PCR was performed on 1–40 ng purified HEV amplicons according to our previous study [32] using 0.4 µM nested PCR primers (Table S1B,D).

### 4.8. Data Analysis

Sequences were assembled using SeqMan Pro v10.1.2 (Lasergene, DNA star 10.1) by aligning fragment sequences against the wbGER27 full HEV genome as reference (GenBank accession number: FJ705359) for HEV0069 and HEV RKI strain (Genbank accession number FJ956757) for HEV0122. Assembled sequences were aligned using the ClustalW Multiple alignment algorithm in BioEdit v7.2.0. Comparison of samples was performed using pairwise nucleotide and amino acid distances. Consensus sequence by assembling multiple forward and reverse reads obtained by Sanger sequencing and detected intra-host variants are reported as alterations. An example electrophoresis plot illustrating the consensus sequence and multiple reads is shown in Figure S3.

Obtained full genome sequences were submitted to Genbank and the accession numbers are: patient HEV0069 feces isolate MN614139 and serum isolate MN6141340; patient HEV0122 feces isolate MN614142 and serum isolate MN614142; cell culture isolate MN614141 and mouse liver isolate MN629976 (Table S2).

## Supplementary Materials

The following are available online at https://www.mdpi.com/2076-0817/8/4/255/s1, Figure S1: HEV viral titers of the sequenced samples and disease course of the patients. Figure S1A serum viral titers of patients HEV0069 and HEV0122, S1B HEV viral load of sequenced feces samples of patients HEV0069 and HEV0122 and liver of human liver chimeric inoculated with feces sample of HEV0069 and S1C viral titers of A549 cell line infected with HEV0069 feces (adapted from [15]). Squares showing the sequenced serum samples and cell culture sample. Figure S2: Schematic representation of size and location of the amplicons of PCR amplification. Figure S3: HEV C3610Y Alteration on Cell Culture Isolate. Table S1: Primer lists and sequences of methods, Table S2: Length of each “full genome” HEV sequence in nucleotides and in reference to wbGER27.
