# Supplementary material for: Hepatitis E Virus Shows More Genomic Alterations in Cell Culture than In Vivo
Source: Pathogens. 2019 Nov 22;8(4):255. doi: 10.3390/pathogens8040255 (PMC6963849; doi:10.3390/pathogens8040255)
Supplement: Supplementary file 1 [file pathogens-08-00255-s001.zip › Supp Materials/Supplementtary Tables.pdf]

pathogens

Hepatitis E virus shows more genomic alterations in cell culture than *in vivo.*

Running title: Spontaneous genomic alterations in HEV

Gulce Sari[1], Martijn D.B. van de Garde[1,*], Anne van Schoonhoven[1,*], Jolanda J.C. Voermans[3], Annemiek A. van der Eijk[3], Robert A. de Man[1], Andre Boonstra[1], Thomas Vanwolleghem[1,2,¶,#], Suzan D. Pas[3,4¶,]

[1]Department of Gastroenterology and Hepatology, Erasmus University Medical Center, Rotterdam, The Netherlands [2]Laboratory of Experimental Medicine and Pediatrics, Faculty of Medicine and Health Sciences, University of Antwerp and Department of Gastroenterology and Hepatology, Antwerp University Hospital, Antwerp, Belgium. [3]Department of Viroscience, Erasmus University Medical Center, Rotterdam, The Netherlands. [4] Microvida, Location Bravis Roosendaal, The Netherlands.

*, [¶] These authors contributed equally to this work.

Corresponding author: TV. Department of Gastroenterology and Hepatology, Erasmus MC – University Medical Center Rotterdam, The Netherlands.

Table-S1A: Primer lists and sequences of method 1[a]

Amplicon nested PCR	Primer name	Template	Sequence 5'- 3'	position[b]	amplicon size (bp)
1.1	FW1[c]	cDNA_RV4	GCAGACCACGTATGTGGTCGATGCC	1	2136
	RV4[c,d]		GTCCGGGTGTACAARGTTCCCTC	2136	
1.2	FW5[c]	cDNA_RV8	TTGTGGCTGCAYCCTGAGGGG	2027	1910
	RV8[c,d]		CAGTGGACTATGTCCGTGAGTTC	3936	
1.3	FW9[c]	cDNA_RV12	TGCCCTGAACTTGAGCAAGGC	3842	2157
	RV12[c,d]		ACCAAACCRGAAGTAGCCTCCTC	5998	
1.4	FW13[c]	cDNA_RV15	TATCGTAACCAGGGYTGGCGCTCTGT	5928	1303
	RV15[c,d]		TTTTTTTTCCAGGGAGCGCG	7230	
1A	FW3[c]	cDNA_RV8	GTTCATATYTGGGACCGGCTYATGCT	974	2062
	RV6[c,d]		CCACYACRTCAACATCCCCCTGCTGTA	3035	
1B	FW7[c]	cDNA_RV12	GCCAACCTAGCACTGGAGATCGATGC	2879	2001
	RV10[c]		CCGGCCGGCAAAGCGCACCACATC	4879	
1C	FW11[c]	cDNA_RV15	GGTTGTATGCTGGCGTGGTGGTGGC	4809	2077
	RV14[c]		CAATCAGAATCTGATCAC	6885	

Table-S1B: Primer lists and sequences for nested PCR of method 1[a]

Amplicon nested PCR	Primer name	Template	Sequence 5'- 3'	position[b]	amplicon size (bp)
1	FW1c	1.1	GCAGACCACGTATGTGGTCGATGCC	1	564
	RV1		CGGGCCATRGCCTCCGCAACATC	564	
2	FW2	1.1	GCCCTGCGGCTAACTGCCGCCG	399	670
	RV2		CTAATCCCGCGGAGGTAGGTCATAAG	1068	
3	FW3c	1.1	GTTCATATYTGGGACCGGCTYATGCT	974	665

	RV3		GGGATGTTAAGYGCCCTATACAG	1638	
4	FW4	1A	GAGGGCTCTGAGGTCGAYCAGGC	1532	605
	RV4c,d		GTCCGGGTGTACAARGTTCCCTC	2136	
5	FW5c	1A	TTGTGGCTGCAYCCTGAGGGG	2027	501
	RV5		GCGYTGGTAGAAGGCATGGCAAAG	2527	
6	FW6	1.2	TTGGCTGGTYAAYGCGTCGAATCC	2458	578
	RV6c,d		CCACYACRTCAACATCCCCCTGCTGTA	3035	
7	FW7c	1.2	GCCAACCTAGCACTGGAGATCGATGC	2879	599
	RV7		TGGACYGTAATCGCACCGGGGTT	3477	
8	FW8	1B	GCGGTCTTTATTCTGGAATGAGCC	3385	552
	RV8c,d		CAGTGGACTATGTCCGTGAGTTC	3936	
9	FW9c	1B	TGCCCTGAACTTGAGCAAGGC	3842	635
	RV9		CCAAGGGAGAAGTTATTTTGRGTGCTATC	4476	
10	FW10	1.3	GACGCATAYGAGGAGTCTGTGTT	4364	516
	RV10c		CCGGCCGGCAAAGCGCACCACATC	4879	
11	FW11c	1.3	GGTTGTATGCTGGCGTGGTGGTGGC	4809	641
	RV11		CGCTGGGAYTGATCACGCCAAG	5449	
12	FW12	1C	CTCCCCTATATTCATCCAACCAACC	5328	671
	RV12c,d		ACCAAACCRGAAGTAGCCTCCTC	5998	
13	FW13c	1C	TATCGTAACCAGGGYTGGCGCTCTGT	5928	575
	RV13		TCATAGTCTTGGATGACCACACG	6502	
14	FW14	1.4	GTCGTCTCAGCCAATGGCGAGC	6372	514
	RV14c		CAATCAGAATCTGATCAC	6885	
15	FW15	1.4	AAACATTCTATGTTCTCCCGC	6775	456
	RV15c,d		TTTTTTTTCCAGGGAGCGCG	7230	

Table-S1C: Primer lists and sequences of 1st PCR round method2 (cDNA from random hexamers)

Amplicon	Primer name	sequence 5'-3'	position[b]	amplicon size (bp)	reference
B1	E3_12S	ACGYATGTGGTCGAWGCCATG	8	995	
	E3_987A	AARAGCATRAGCCGRTCCCA	1002		
B2	E3_561S	ATGGCCCGICAYGGGATGAC	557	1550	
	E3_2091A[g]	AAIGGRTTIGCIGAYTCCCA	2106		
B3	E3_22S[g]	TCGAWGCCATGGAGGCCCA	18	1582	
	E3_1584A[g]	CCRTGRACIGCRTARGTCCC	1599		
B4	E3_1527S[g]	GGCYTAYGARGGITCIGARGT	1525	1254	
	E3_3025A	GGGCGRTGRTTRCGYTCCCA	2778		
B5	E3_2032S[g]	TRTGGYTRCAYCCYGAGGG	2028	1507	
	E3_3781A[g]	CTRGCRTCRGCYGTRGCTAT	3534		
B6	E3_3475S	CTYGGYGAYCCIAAYCAGAT	3209	1781	
	E3_5236A	ACATCRACACARACCTGCGC	4989		
B7	E3_4759S	ATGGARGARTGYGGYATGCC	4493	861	Muñoz-Chimeno, et al.

	E3_5597A	GGGTTGGTTGGATGAATATAGGGG	5353		
B8	E3_5236S	TGCCTATGYTGCCCGCGC	5212	1189	
	E3_6648A	ACWGYYGGCTCACCATTGGC	6400		
B9	E3_6474S	GCIGCYACRCGITTYATGAA	6204	922	
	E3_7371A	TTYTAAGRCGCTGAAGYTCAG	7125		
B8x[f]	M8x-F (E3_5227**S**)	ACGAAYGTIGCGCAGGTYTG	4961	1440	adapted from Muñoz-Chimeno, et al.
	E3_6648A	ACWGYYGGCTCACCATTGGC	6400		Muñoz-Chimeno, et al.
1.4[f]	FW13	TATCGTAACCAGGGYTGGCGCTCTGT	5928	1303	this study
	RV15	TTTTTTTTCCAGGGAGCGCG	7230		
1C[f]	FW11	GGTTGTATGCTGGCGTGGTGGTGGC	4809	2077	
	RV14	CAATCAGAATCTGATCAC	6885		

Table-S1D: Sequences and details of primers used for nested PCR amplification method 2 and Sanger sequencing of resulting fragments.

Amplicon nested PCR	Primer name	Template	Sequence 5'- 3'	position[b]	amplicon size (bp)	reference
B1n	E3_22Sg	B1	TCGAWGCCATGGAGGCCCA	18	556	Muñoz-Chimeno, et al.
	E3_561A		GTCATCCCRTGICGRGCCAT	573		
B2n	E3_984S	B2	ATYTGGGAYCGGCTYATGCT	980	620	
	E3_1584Ag		CCRTGRACIGCRTARGTCCC	1599		
B3n	E3_120S	B3	GCTGTGGTGGTYCGGCCGTT	113	1217	
	E3_1314A		CGRCAYTGIGCRTARAACTG	1329		
B4n_1	E3_1527Sg	B4	GGCYTAYGARGGITCIGARGT	1525	582	
	E3_2091Ag		AAIGGRTTIGCIGAYTCCCA	2106		
B4n_2	E3_2032Sg	B4	TRTGGYTRCAYCCYGAGGG	2028	649	
	E3_2923A		GTYTCICGRTAYGCYGCCTC	2676		
B5n_1	E3_2896S	B5	AGGRTYGARCAGAAYCCIAAGAG	2632	683	
	E3_3563A		CGRTGIGTIACRTGCCACCA	3314		
B5n_2	E3_3316S	B5	GAGCTYCGIAAYAGYTGGCG	3051	484	
	E3_3781Ag		CTRGCRTCRGCYGTRGCTAT	3534		
B6n_1	E3_3592S	B6	GAYGTITGYGARCTYATACG	3326	812	
	E3_4384A		TCCTGRCCCTTYTCIACCAT	4137		
B6n_2	E3_4192S	B6	ATAGTYCAYTGYCGIATGGC	3926	1055	
	E3_5227A		CARACCTGCGCIACRTTCGT	4980		
B7n	E3_4895S	B7	GGAAYACYGTYTGGAAYATGGC	4629	722	
	E3_5592A		TTGGTTGGATGAATATAGGGGAGGG	5350		

B8n	E3_5478S	B8x	TGCCTATGYTGCCCGCGC	5209	1175	
	E3_6631A		GGCYGARACRACIGGGCGGG	6383		adapted from Muñoz-Chimeno, et al.
B9n	E3_6587S	B9	CCGACAGAATTRATTTCGTCGGC	6321	735	Muñoz-Chimeno, et al.
	E3_7302A		TCMGGRCARAAATCATCRAAAGT	7055		
6.3nf	M8x-F (E3_5227S)	B6	ACGAAYGTIGCGCAGGTYTG	4961	390	adapted from Muñoz-Chimeno, et al.
	E3_5592A		TTGGTTGGATGAATATAGGGGAGGG	5350		Muñoz-Chimeno, et al.
8.1nf	E3_5478S	B8x	TGCCTATGYTGCCCGCGC	5209	790	Muñoz-Chimeno, et al.
	RV12		ACCAAACCRGAAGTAGCCTCCTC	5998		this study
8.2nf	FW13	B8x	TATCGTAACCAGGGYTGGCGCTCTGT	5928	456	this study
	E3_6631A		GGCYGARACRACIGGGCGGG	6383		adapted from Muñoz-Chimeno, et al.
2f,h	FW2	B3	GCCCTGCGGCTAACTGCCGCCG	399	670	this study
	RV2		CTAATCCCGCGGAGGTAGGTCATAAG	1068		
13f,h	FW13	1C	TATCGTAACCAGGGYTGGCGCTCTGT	5928	575	
	RV13		TCATAGTCTTGGATGACCACACG	6502		
15f,h	FW15	1.4	AAACATTCTATGTTCTCCCGC	6775	456	
	RV15		TTTTTTTTCCAGGGAGCGCG	7230		

[a] reference: this study, [b] according to Genbank accession FJ705359, [c] primers used in first and nested PCR method 1, [d] Primers used for cDNA synthesis method 1, [e] primers also used in first and nested PCR method 1, [f] additonal PCRs with reference to Muñoz-Chimeno, et al. [g] primers used in first and nested PCR method 2, [h] amplicons used in nested PCR method 1

Table-S2. Length of each “full genome” HEV sequence in nucleotides and in reference to wbGER27.

Sample	Sequence length (nt)	Fraction of full genome (%)	Range on wbGER27	Nucleotide identity to wbGER27*	Genbank Accession Number
HEV0069 serum	7189	99.6%	13 - 7202	96.8%	MN6141340
HEV0069 faeces	7110	98.3%	12 - 7122	96.8%	MN614139
HEV0069 A549 cell culture supernatant	7232	100%	1 - 7232	96.7%	MN6141341
HEV0069 mouse liver	7110	98.3%	12-7122	96.7%	MN629976
HEV0122 serum	6431	88.9%	29 - 5346, 5951 - 7059	81.1%	MN614143
HEV0122 faeces	6179	85.4%	23 - 5097, 5951 - 7056	80.5%	MN614142

*Pairwise nucleotide identity was calculated over the indicated range of each obtained sequence.
