# Supplementary figures and images for: Hepatitis E Virus Shows More Genomic Alterations in Cell Culture than In Vivo
Source: Pathogens. 2019 Nov 22;8(4):255. doi: 10.3390/pathogens8040255 (PMC6963849; doi:10.3390/pathogens8040255)

Figure-S1

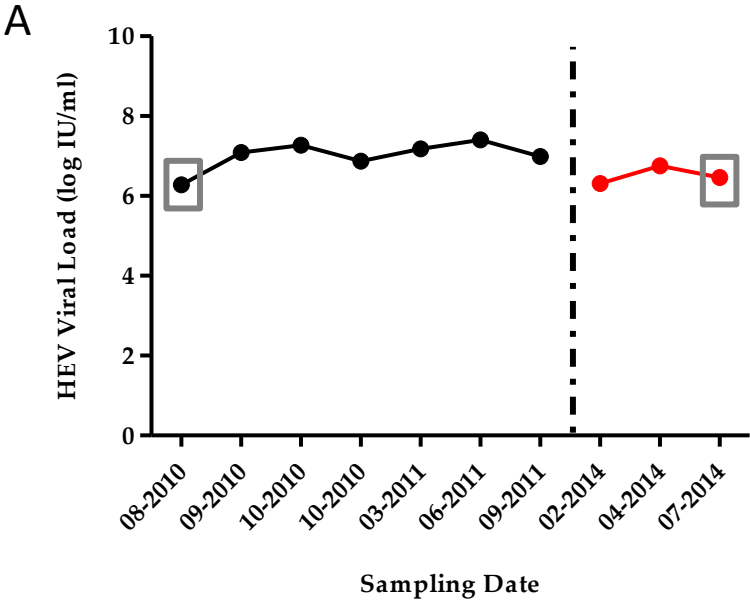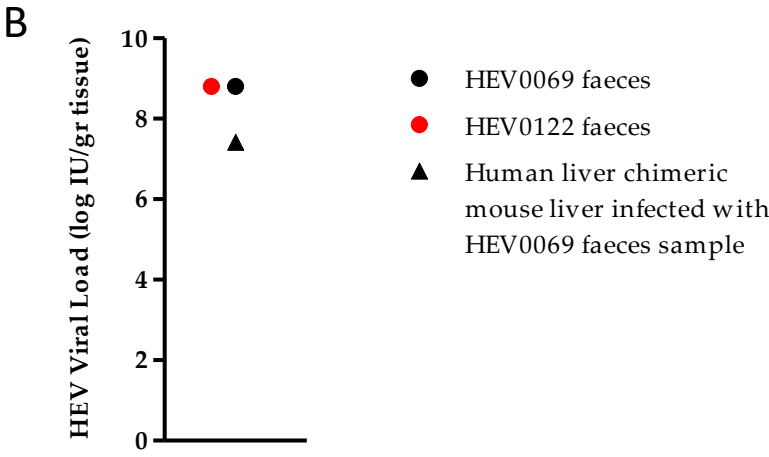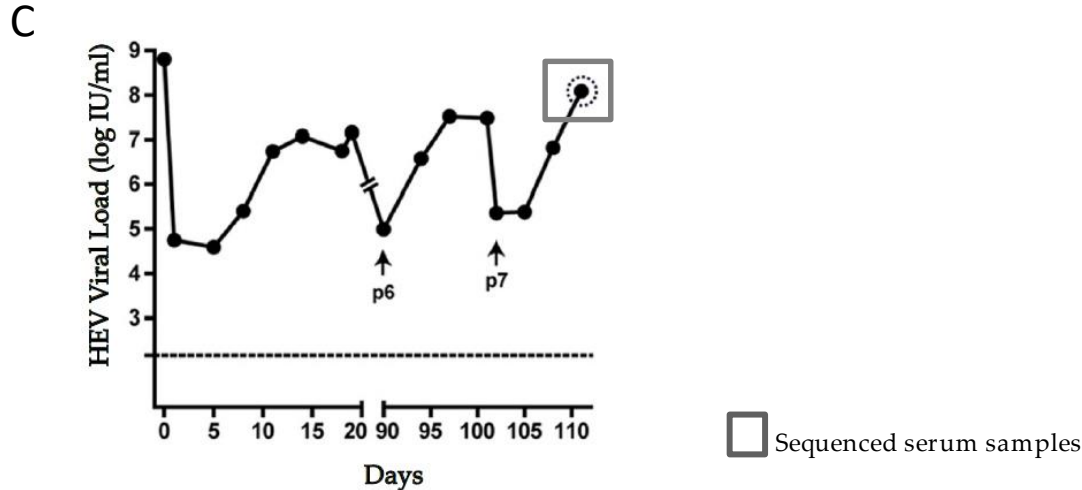

Figure-S2

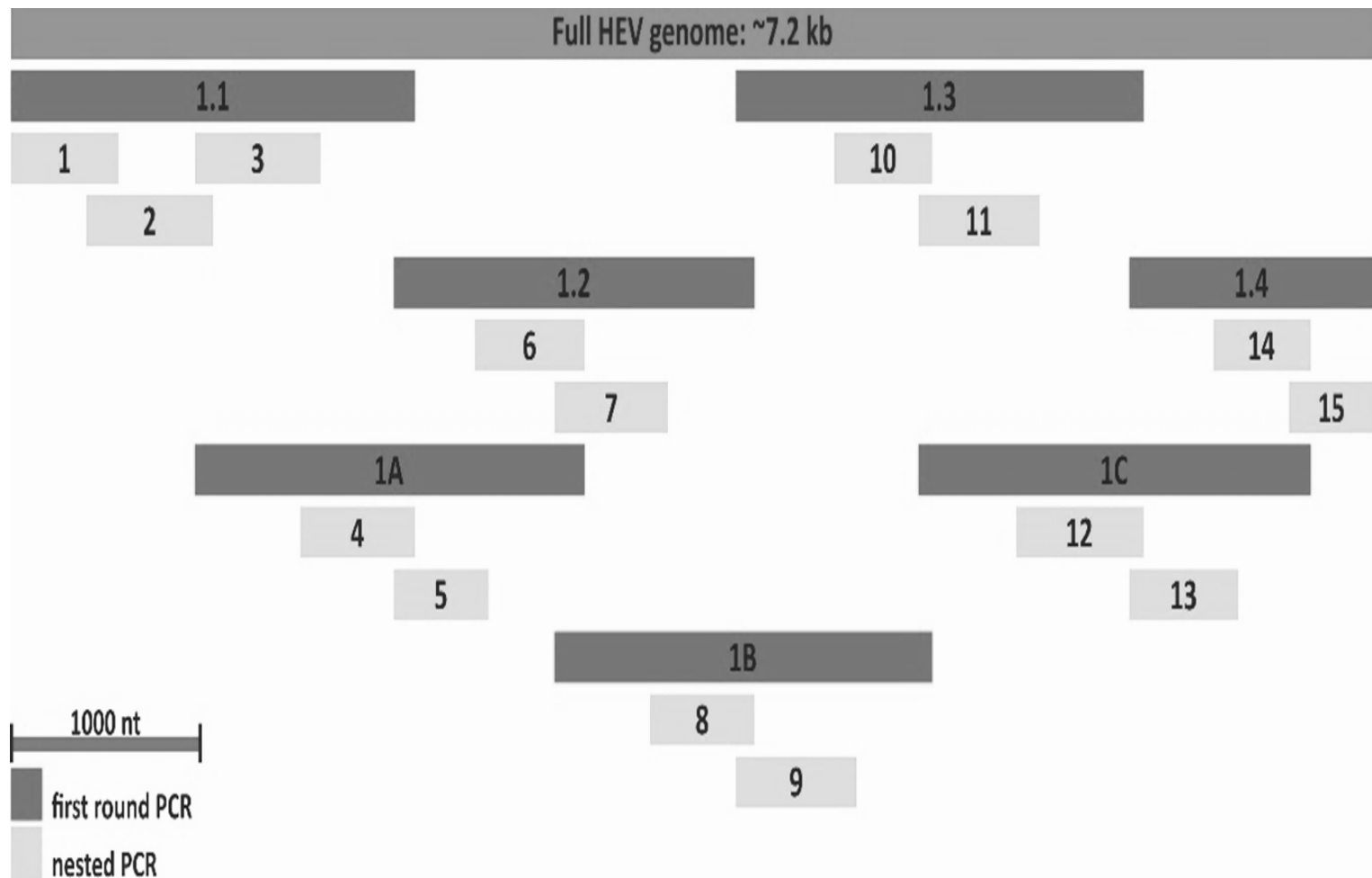

Figure-S3

HEV C3610Y Alteration in Cell Culture Isolate

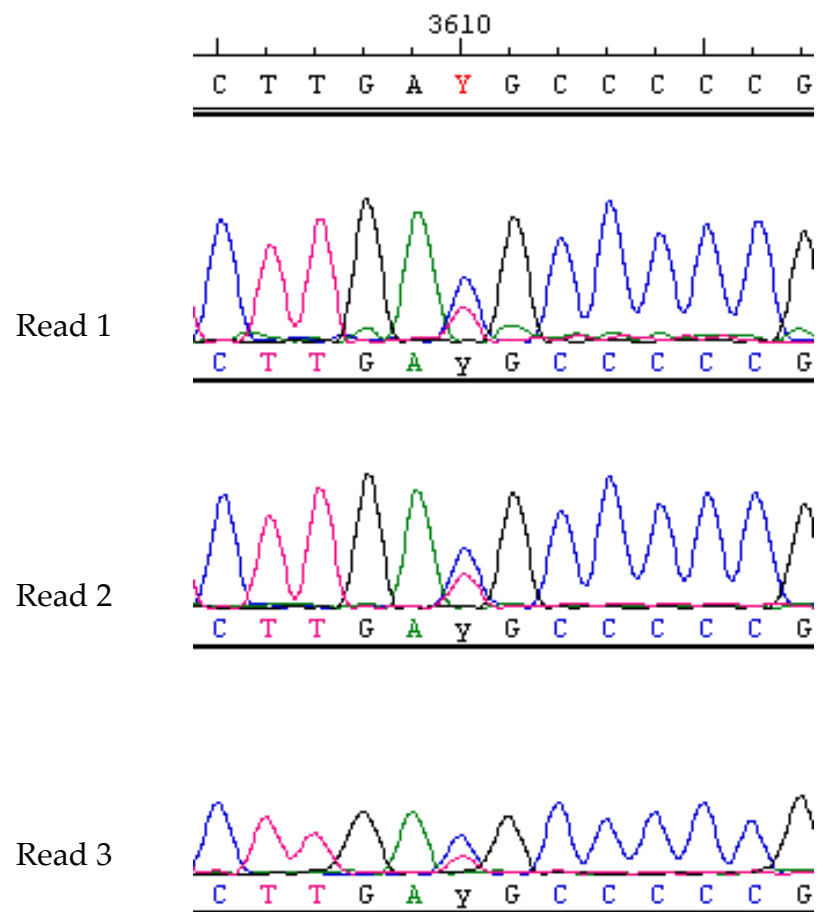

Supplement: Supplementary file 1 [file pathogens-08-00255-s001.zip › Supp Materials/Supp Figures.pdf]
